# How and When Are Job Crafters Engaged at Work?

**DOI:** 10.3390/ijerph15102138

**Published:** 2018-09-28

**Authors:** Inyong Shin, Won-Moo Hur, Seongho Kang

**Affiliations:** 1 College of Business Administration, Pukyong National University, 45 Yongso-ro, Nam-gu, Busan 48513, Korea; shiny@pknu.ac.kr; 2 College of Business Administration, Inha University, 100 Inha-ro, Michuhol-gu, Incheon 22212, Korea; wmhur@inha.ac.kr; 3 College of Business, Chosun University, 309 Pilmundae-ro, Dong-gu, Gwangju 61452, Korea

**Keywords:** work engagement, job crafting, psychological capital, coworker support

## Abstract

The importance of work engagement and the lack of engaged employees have led researchers to focus on how to enhance employees’ levels of engagement in the workplace. Although job crafting as a principal driver of work engagement has recently received much attention from academics, little is known about the processes and conditions in which employees who craft their tasks become engaged. In order to address these research gaps, we hypothesize that psychological capital (PsyCap) is likely to mediate the association between job crafting and work engagement, and that coworker support, rather than supervisor support, is likely to moderate the relationship between job crafting and PsyCap. Further, we integrated these hypotheses and tested the moderated mediation effect. Using survey data from 175 flight attendants in South Korea, we found the results to be in line with our expectations. The findings of this empirical research contribute to the understanding of how and when job crafters become engaged at work.

## 1. Introduction

In the midst of a rapidly changing business environment, contemporary corporate organizations continue to seek ways to increase their viability and prosperity. Scholars and practitioners generally agree that the existence of engaged members within an organization seems to offer a solution to these challenges. As engaged workers are inclined to be enthusiastically connected to their work activities and to deal with work demands well [1,2], they tend to produce constructive outcomes for their organizations. For instance, meta-analytic reviews on work engagement have revealed that it contributes not only to the enhancement of task and contextual performance at the individual level, but also to business performance, such as productivity and profitability, at the organizational level [3,4]. Meanwhile, a global workforce study conducted by a consulting firm indicated that organizations with highly engaged members obtain operating margins that are more than 5% higher than those with highly disengaged members [5]. In addition to these beneficial work outcomes, work engagement is regarded as an indicator of employee health and well-being by virtue of the fact that engaged workers stay mentally healthy and gain the health-related ability that is required for managing their tasks [2,6]. Nonetheless, in reality, few employees are actually engaged at work. According to a survey by a research institute, the average percentage of engaged employees across the globe is a surprisingly low 13%, a number that falls to just 6% in East Asia [7].

The importance of work engagement and the lack of engaged employees have led researchers to focus on how to enhance employees’ levels of engagement in the workplace. Previous studies have found that a variety of factors, including individual characteristics [4], job resources [2,6,8], organizational resources [1], and leadership styles [4], serve as antecedents of work engagement. In particular, some researchers have recently noted that workers have a tendency to proactively shape the boundaries of their jobs, revealing that, when employees craft their jobs, they become engaged in their work (e.g., [9,10,11]). Despite valuable findings that actions taken by individuals in order to create changes in the course of their work determine the level of their work engagement, there remain unresolved issues that might have significant implications for the work engagement research. First, little is known about how job crafting has an effect on work engagement. In this regard, Chen and colleagues [12] (p. 22) argue that investigating whether job crafting affects work engagement without understanding the underlying mechanisms between the two might be assertive and arbitrary. Second, as yet we do not have much knowledge on when the association between job crafting and work engagement is strengthened or weakened. Although the link between job crafting and work engagement might depend on the neighboring circumstances in which employees work together, previous research has neglected exploring this possibility.

In order to address these research gaps, we seek to identify how and when job crafters become engaged in their work, with a particular focus on personal and job resources. Specifically, given that work engagement inherently represents the active investment of personal resources in work experience [4,13,14], we suggest that psychological capital (PsyCap) as a personal resource will play a mediating role in which job crafting relates to work engagement. We also posit that the association between job crafting and PsyCap will vary depending on social support, a job resource, such that employees who craft their jobs will gain more PsyCap, provided that they receive much social support. Social support is deemed suitable for this study, given the moderating role of social support on the relationships of job aspects with employee outcomes, as confirmed by the job demands-control-support model that has been influential in the field of occupational health research [15]. Yet, while considering that employees often alter their tasks without the approval from their supervisors and that they are more frequently exposed to their fellow workers than their supervisors [16,17], we expect that coworker support rather than supervisor support will play a moderating role in the link between job crafting and PsyCap. We further propose a model that incorporates a moderated mediation role for coworker support that fortifies the mediation role of PsyCap on the job crafting-work engagement association.

In the remainder of this study, we begin by reviewing the concepts of job crafting and work engagement, and then positing that job crafting relates positively to work engagement. We subsequently look into the mediating potential of PsyCap and the amplifying potential of coworker support in order to fully understand the link between job crafting and work engagement, before integrating these into the moderated mediation model. Next, we consider whether our expectations were reasonable on the basis of the analysis of the data obtained from employees. Finally, we discuss the implications and limitations arising from this study and make suggestions for further research.

## 2. Theoretical Review and Hypothesis Development

### 2.1. Employees’ Job Crafting and Work Engagement

In terms of job design, a top-down approach in which tasks that are designed and structured by an organization are assigned to its members, such as the job characteristics model [18], has traditionally been pervasive. However, as the turbulence of business environments has increased, the perspective on job design has changed [19]. In conditions of high uncertainty, it is almost impossible for managers to forecast all contingencies in advance and to formalize specific tasks for employees with any precision [20]. Instead, since Wrzesniewski and Dutton [21] introduced the notion of job crafting as a self-initiated form of behavior in which employees proactively shape the boundaries of their jobs so as to align them with their own preferences and abilities, a bottom-up approach has emerged whereby job incumbents play an active role in altering their own tasks for themselves.

A representative version of the bottom-up approach is known as job crafting, conceptualizations of which have evolved into two major streams. On the one hand, the introducers of job crafting define it as “the physical and cognitive changes individuals make in the task or relational boundaries of their work”, delineating the types of job crafting as task crafting (“altering the form or number of activities one engages in while doing the job”), relational crafting (“exercising discretion over with whom one interacts while doing the job”), and cognitive crafting (“altering how one sees the job”) [21] (pp. 179–180). On the other hand, while expanding the job demands-resources (JD-R) model, some scholars describe job crafting as “the changes that employees may make to balance their job demands (all aspects of the job that require sustained physical and/or psychological effort or skill) and job resources (those aspects of the job that are either/or functional in achieving work goals) with their personal abilities and needs” [22] (p. 174). The latter further identify the forms of job crafting as “increasing structural job resources”, “increasing social job resources”, and “increasing challenging job demands”, and “decreasing hindering job demands” [22] (p. 174). In a similar vein, others classify job crafting into “decreasing hindering job demands”, “increasing social job resources”, “increasing challenging job demands”, and “increasing structural job resources” [23] (p. 367), and categorize it into “increasing challenging demands”, “decreasing social demands”, “increasing social job resources”, “increasing quantitative demands”, and “decreasing hindering demands” [24] (p. 376).

Meanwhile, work engagement is described as “a positive, fulfilling, work-related state of mind that is characterized by vigor (high levels of energy and mental resilience while working), dedication (a sense of significance, enthusiasm, inspiration, pride, and challenge), and absorption (being fully concentrated and deeply engrossed in one’s work, whereby time passes quickly)” [25] (pp. 74–75) As employees become more engaged, they approach their work more energetically, enthusiastically, and engrossedly, and in turn, expand the view of their roles and take on a broader set of activities in the workplace [1,26]. As a result, when compared to disengaged employees, engaged workers more frequently exhibit in-role, extra-role, and proactive behaviors [14,27,28], and show lower turnover intention [29]. Organizations that retain engaged members achieve superior business performance in terms of profitability and productivity [3]. Additionally, engaged employees enjoy good mental health instead of experiencing symptoms, such as distress and depression [2].

Researchers interested in the association between job crafting and work engagement have found that job crafters tend to become engaged in their work (e.g., [9,10,11]). Yet, some scholars studying job crafting based on the expanded JD-R model have yielded results that differed from those that were expected for the relationships between the types or forms of job crafting and work engagement. For example, increasing social job resources, decreasing social job demands, and decreasing hindering job demands have, in instances, been found to be unrelated to work engagement [23,24], and decreasing hindering job demands has even been identified as being negatively related to work engagement [22]. We thus attempt to investigate the relationship between job crafting and work engagement based on the definition by Wrzensniewski and Dutton [21] in accordance with the original concept of job crafting. In particular, this study focuses on the task facet of job crafting, because it has been recognized as the main form of job crafting in the previous research (e.g., [30]). Assuming that job crafting is likely to have a positive relationship with work engagement, we examine the mediating role of PsyCap and the moderating role of coworker support in the relationship below.

### 2.2. Mediating Role of Psychological Capital

Luthans and colleagues originated the construct of PsyCap to capture one’s psychological capacities and personal resources, defining it as “an individual’s positive psychological state of development” that contains the following four resources: “self-efficacy (having confidence to take on and put in the necessary effort to succeed at challenging tasks)”, “hope (persevering toward goals and, when necessary, redirecting paths to goals)”, “optimism (making a positive attribution about succeeding now and in the future), and resilience (when beset by problems and adversity, sustaining and bouncing back and even beyond)” [31] (p. 3). As PsyCap inherently has a malleable and developmental property, it has the potential to be enhanced through training or practice [32,33]. PsyCap also has been found to influence various worker outcomes, such as attitudes, behavior, performance, and well-being [34,35]. In this way, PsyCap might not only be developed and improved, but it could also affect employees’ work lives. Further, work engagement reflects the active investment of one’s personal resources in conducting his or her work [4,13,14]. Accordingly, we believe that PsyCap is suitable for the expectation of this study that PsyCap will play the mediating role in linking job crafting with work engagement.

We firstly anticipate that when workers craft their tasks they will feel enhanced PsyCap. Specifically, verifying employees’ capabilities with regard to new work-related activities in their work environment could benefit mastery experiences, resulting in higher levels of self-efficacy [36]. Jobs that are enriched by crafting individuals’ task boundaries are also likely to improve their abilities to develop or identify alternative pathways to reach their goals, which would consequently foster their hope [37]. Moreover, job crafters should maintain a positive evaluation of various events occurring in the workplace by exercising control over their work conditions, and as a result, experience increased the levels of optimism [11]. Lastly, job crafting tends to enable the ability to handle and overcome difficult work situations, which would be helpful to employees in terms of building or improving their resilience [38]. Based on this reasoning, it is expected that job crafting will positively influence PsyCap comprising hope, optimism, self-efficacy, and resilience. This study further predicts that workers with higher levels of PsyCap are more likely to engage in their work. In practice, this means that employees with high PsyCap are inclined to be confident in their competencies to perform the tasks, to create possible means and alternatives to accomplish those tasks, to believe that they will obtain beneficial work outcomes, and to adapt to challenging work situations [39,40]. The benefits arising from these personal resources have been reported to make a great contribution to their energetic, enthusiastic, and engrossed approach to the work, as well as the quality of their working lives [8,41].

In sum, if employees craft their tasks, they become self-efficacious, optimistic, hopeful, and resilient, and eventually, they are likely to be vigorous, dedicated, and absorbed in their work. In other words, it is expected that job crafting will foster PsyCap, which in turn will promote work engagement. Accordingly, we suggest the following hypothesis:

**Hypothesis** **1** **(H1).** *Psychological capital mediates the positive association between job crafting and work engagement.*

### 2.3. Moderating Role of Coworker Support

Social support from useful social interaction with people in the workplace is considered to be one of the job resources that is beneficial to one’s work conduct and personal functioning [42]. Researchers generally recognize that employees’ experiences in performing their work are greatly affected by their acquisition of social support with the accumulated evidence, indicating that social support functions to protect against the harmful impacts of job demands [15,43]. It is thus expected that there seems to be a complementary association between job crafting and social support. That is, as job crafters receive social support from others in the workplace, their levels of PsyCap will become higher.

Yet, we notice that since social support within an organization is to be found horizontally from coworkers and vertically from supervisors, it can be divided into coworker support and supervisor support. Further, we posit that coworker support rather than supervisor support will function as a critical boundary condition that links job crafting to PsyCap for the following two reasons. First, job crafting usually takes place at work without the knowledge or approval of supervisors [16,17]. That is, employees are inclined to alter work-related procedures or the scope of their work according to their own judgment. Therefore, it is probable that the influence of supervisors on employees’ job crafting efforts or the results of those endeavors is negligible. Second, as compared to supervisor support, coworker support tends to be more valued and consistent, and less influenced by organizational politics [44]. As coworkers comprise the majority of ‘others’ with whom a job incumbent interacts during his or her work, he or she is more frequently exposed to coworkers than to supervisors [45]. Under this condition, the sources of meaningful social support for job crafters seem to be not supervisors but coworkers.

Supportive work environments that derive from helpful coworkers provide an employee with work-related assistance, informative advice, and emotional empathy [45]. Such instrumental and emotional help in the workplace leads the employee to drive for new challenges, to experiment, to make changes, and to embrace their own creativity [46]. Accordingly, the support that a worker receives from his or her coworkers is expected to have a significant impact on the continuance and outcome of the efforts that the focal worker makes regarding job crafting. In addition, coworker support enables a job incumbent to feel that his or her personal resources are not subject to loss or insufficiency [47]. When employees craft their tasks under conditions of high coworker support, they are more likely to feel that they have abundant personal resources. With the above discussion in mind, we propose the following hypothesis:

**Hypothesis** **2** **(H2).** *Coworker support amplifies the positive association between job crafting and psychological capital, such that the association is larger among employees with high coworker support than those with low coworker support.*

### 2.4. Moderated Mediation Model

**Figure 1:**
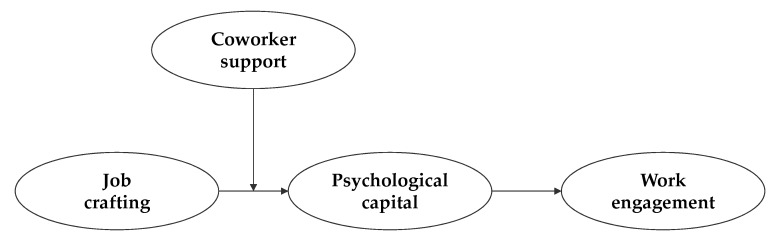
Research model.

The current study proposes a moderated mediation model to comprehensively account for the association between job crafting and work engagement, whereby the support that is garnered from coworkers tends to reinforce the indirect link between job crafting and work engagement through PsyCap. In this scenario, the personal resources of job crafters who receive support from their coworkers are likely to increase, which then boosts their work engagement. Therefore, we expect the following moderated mediation relationships and suggest a research model that reflects this expectation in Figure 1:

**Hypothesis** **3** **(H3).** *Coworker support amplifies the positive and indirect association between job crafting and work engagement through psychological capital, such that the association is larger among employees with high coworker support than those with low coworker support.*

## 3. Method

### 3.1. Data Collection and Sample Characteristics

For the purposes of this study, we collected data from flight attendants that were working for an airline in South Korea. Flight attendants who craft their own jobs proactively, instead of relying on supervisors’ orders, are more likely to improve customer service quality and their work engagement [12]. In this regard, cabin crews have received much interest in the literature on job crafting and work engagement, and they have been considered to be suitable for testing the research model of this study.

We first contacted all five small-sized airlines in South Korea. Of them, only one airline company agreed to participate in our research. Having contacted the human resource (HR) managers of this airline for gathering the data, we had the HR managers administer a survey to the flight attendants, each of whom received a self-administered questionnaire, and a stamped addressed envelope, and instructions to seal and return the completed questionnaire directly to the researchers in order to ensure confidentiality.

The HR managers of the airline recruited research participants using e-mail and company bulletin board. A total of 273 employees applied for the participation of this research project. Of 273 questionnaires that were delivered to the survey participants, 175 questionnaires were returned, leading to a response rate of 64.8%. We employed the full-information maximum likelihood (FIML) estimation technique to deal with missing values (missing data points: about 5.7–6.9% by variable). The FIML method is desirable in that other alternative statistical approaches (e.g., listwise-deletion) might yield biased results [48]. The average age and job tenure of the participants, of whom 89.1% were women, were 28.5 (SD = 3.9) years and 4.9 years (SD = 4.2) respectively, whilst their educational attainment varied, as follows: 8.1% two-year vocational college, 84.9% four-year university, and 7.0% graduate school. To test the equivalence of our sample and those who did not participate in our study, we requested HR managers to compare the demographic characteristics of the respondents and overall employees. They confirmed that there were no significant differences between the two groups in terms of gender, age, organizational tenure, and education. This finding suggests that our sample is well representative of the overall employees at the sponsoring company.

### 3.2. Measurement Scales

Brislin’s [49] back-translation method was adopted for the purposes of this study, whereby the original survey items were first translated into Korean before being back-translated into English. Once this was done, four bilingual experts were asked to review the back-translated version to ensure that the survey items were identical to the original items, thereby confirming that the Korean version was acceptable for use. 

All of the measurement items utilized a five-point Likert-type scale. Job crafting was measured by four items that were borrowed from Slemp and Vella-Brodrick’s [50] task crafting scale (e.g., “I change the scope or types of tasks that I complete at work”), which has been recognized as the core of job-crafting activities in the previous research (e.g., [30]). PsyCap was measured using 24 items (four sub-dimensions: efficacy: “I feel confident analyzing a long-term problem to find a solution”; hope: “At the present time, I am energetically pursuing my work goals”; optimism: “I always look on the bright side of things regarding my job”; resilience: “I can be ‘on my own’, so to speak, at work if I have to”) adapted from Luthans et al. [51]. For the continuous latent construct of PsyCap, we followed the guidelines that were suggested by Russell et al. [52] to create three items each for the four sub dimensions of PsyCap. Specifically, an exploratory factor analysis was performed on items within the PsyCap scales, and “they were then assigned to parcels that were based on item factor loadings as a way to balance the averaged loadings across parcels” [52]. To assess the extent to which an individual is engaged in his or her work, we utilized the nine-item Utrecht Work Engagement Scale (UWES-9; [53]) (e.g., vigor: “I felt bursting with energy”; dedication: “I was enthusiastic about my job”; absorption: “I felt happy when I was working intensely”). As a measure of coworker and supervisor support, we used four items from Tews, Michel, and Ellingson’s [54] social support scale (e.g., “My coworkers/supervisor go out of their way to help me with work-related problems”).

We controlled for age, gender, education, job tenure (years), and the Positive and Negative Affect Schedule (PANAS) in all analyses. These covariates were controlled for because they were shown to influence levels of PsyCap (e.g., [55,56]) and work engagement (e.g., [28,57]). To evaluate PANAS, we used three items each from The International Positive and Negative Affect Schedule Short Form [58]. We requested participants to report the extent to which they generally experience positive affect (i.e., “inspired, attentive, and active”) and negative affect (i.e., “upset, hostile, and nervous”). 

## 4. Results

### 4.1. Test of Measurement Model

The reliability of the constructs was judged while using Cronbach’s alpha (see Table 1). Their reliability coefficients ranged from 0.81 to 0.90, exceeding the criterion of 0.70 [59]. Next, we conducted a confirmatory factor analysis (CFA) using M-plus 8.1 software to test the convergent and discriminant validity of the measurement items (χ^2^_(637)_ = 1017.38; *p* < 0.05, the comparative fit index [CFI] = 0.91, the Tucker-Lewis index [TLI] = 0.90, the root-mean-square error of approximation [RMSEA] = 0.06, the standardized root-mean-square residual [SRMR] = 0.07). All of the constructs showed strong reliability, with composite reliability (CR) ranging from 0.81 to 0.91. Finally, we assessed the discriminant validity among the constructs [60]. All of the average variance extracted (AVE) values were larger than the squared correlations between specific constructs and any others. These results indicated that the constructs included in this study possessed sufficient reliability and validity. 

Because all of the research data were examined cross-sectionally by self-report, it was required to test for potential biases resulting from common method variance (CMV). Following Podsakoff et al.’s [61] guidelines, we built in several precautions to reduce CMV (e.g., securing respondent anonymity and separating the measurement of predictor, mediator/moderator, and final outcomes). In addition, we performed Harman’s one-factor analysis. All of the fit indices demonstrated a worse fit for the single-factor model compared to the measurement model of this study (χ^2^_(665)_ = 2777.91; *p* < 0.05, CFI = 0.49, TLI = 0.46, RMSEA = 0.14, SRMR = 0.11).

We also employed an additional latent common method factor (LCMF) to the measurement model to test the size of method effect [61]. This factor did not account for any substantial variance in the indicator variables (1.3%), as an average of 18–32% of the variance in a certain measure is attributable to method variance [61]. Furthermore, the standardized factor loadings of all items were below 0.3 for the LCMF and were statistically not significant. Taken together, we judged that our data were not seriously influenced by CMV.

### 4.2. Test of Hypotheses

Hypothesis 1 proposed a mediation effect model in which the positive association between job crafting and work engagement would be mediated by PsyCap. To verify this hypothesis, we firstly employed a regression analysis and estimated two coefficients (see Table 2). The results showed that controlling for gender, age, education, job tenure, and PANAS, job crafting was positively related to PsyCap (*b* = 0.21, *p* < 0.01). Further, controlling the covariates and job crafting, PsyCap was found to be positively associated with work engagement (*b* = 0.57, *p* < 0.01). To estimate the mediation effect, we used bootstrapping (*N* = 5000), which is a statistical resampling method that estimates the standard deviation of a model from a sample [62]. As shown in Table 3, the results indicated that, when controlling for gender, age, education, job tenure, and PANAS, the indirect effect of job crafting on work engagement via PsyCap was significant (*b* = 0.12, 95% CI [0.06, 0.19]). When PsyCap was included in the model, the direct effect of job crafting on work engagement was still significant (*b* = 0.19, 95% CI [0.06, 0.34]). These results implied that the association between job crafting and work engagement was partially mediated by PsyCap. Based on the results of the regression analysis and bootstrapping, we found that Hypothesis 1 was accepted.

Prior to the moderation and moderated mediation analyses, all continuous variables were mean-centered [62]. In Hypothesis 2, we predicted that coworker support would moderate the association between job crafting and PsyCap. As revealed in Table 4, coworker support strengthened the positive association between job crafting and PsyCap (*b* = 0.19, *p* < 0.05). Nonetheless, it was found that the association between job crafting and PsyCap was not significantly moderated by supervisor support (*b* = −0.07, *p* > 0.05).

**Figure 2:**
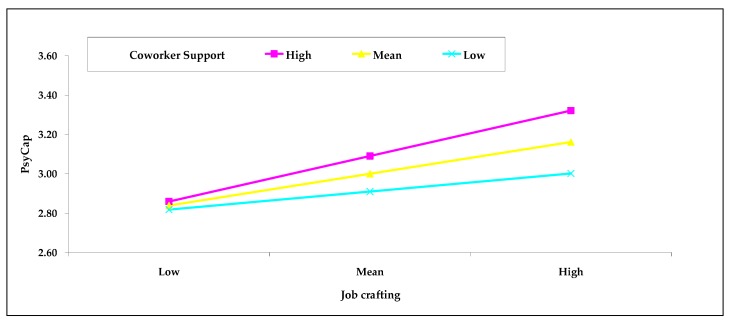
Relationship between job crafting and PsyCap moderated by coworker support.

In addition, as is suggested in Table 5 and Figure 2, a subsequent simple slope test (graphing simple slopes at ±1 SD of coworker support) showed that the positive association between job crafting and PsyCap was more apparent among employees with high and average levels of coworker support (high: *b* = 0.28, 95% CI [0.16, 0.40]; average: *b* = 0.18, 95% CI [0.09, 0.28]). In contrast, job crafting was not assoiciated with PsyCap when employees experienced a low level of coworker support (low: *b* = 0.09, 95% CI [−0.06, 0.24]). Therefore, Hypothesis 2 was accepted.

Hypothesis 3 proposed a moderated mediation model. To confirm this hypothesis, we estimated the conditional mediation effect of job crafting on work engagement through PsyCap, depending on the levels of coworker support. The conditional mediation effect of job crafting on work engagement via PsyCap was strengthened by coworker support (*b* = 0.11, 95% CI [0.004, 0.219]). Further, as shown in Table 6, the mediation effect of job crafting on work engagement through PsyCap was positively significant for the high and average levels of coworker support (high: *b* = 0.16, 95% CI [0.09, 0.25]; average: *b* = 0.10, 95% CI [0.05, 0.17]). However, when the level of coworker support was low, the indirect effect of job crafting on work engagement through PsyCap was not significant (low: *b* = 0.05, 95% CI [−0.03, 0.15]). Therefore, Hypothesis 3 was accepted.

## 5. Discussion

Although researchers have been interested in job crafting as one of the principal drivers of work engagement, they have tended to neglect exploration of the issues regarding how and when job crafting relates to work engagement. To address these research gaps, the current study sought to pinpoint mediators and moderators in the relationship between job crafting and work engagement, developing and testing a research model to comprehensively understand the relationship between them, which incorporated PsyCap as the mediator and coworker support as the moderator. Data from 175 employees from an airline company supported our expectations, revealing a link between job crafting and work engagement that is mediated by PsyCap, whereby the relationship between job crafting and PsyCap was moderated by coworker support (but not supervisor support), and the indirect relationship between job crafting and work engagement through PsyCap was moderated by coworker support (but not supervisor support).

### 5.1. Theoretical Contributions and Practical Implications

This study makes a number of contributions to the relevant literature. Having noted that prior scholarly interest in the relationship between job crafting and work engagement (e.g., [9,10,11]) has done little to clarify the relationship, the one exception being attempted to elucidate an underlying mechanism utilizing person-job fit theory [12], we reveal a new mediator in the link between job crafting and work engagement whereby employees who craft their tasks are more likely to gain PsyCap and thereby become more engaged in their work. Some studies considered PsyCap as a variable moderating the relations between predictors (e.g., leadership styles and organizational politics) and outcomes (e.g., employee attitudes and behavior) [63,64]. In this study, however, we have paid our attention to the possibility that an employee’s level of PsyCap is likely to be determined by his or her degree of job crafting, and attempted to verify that PsyCap functions as a variable bridging job crafting and work engagement. In particular, moving beyond previous research that focused on the direct effects of job crafting on PsyCap and work engagement [11], the current study suggests the theoretical rationale and empirical evidence for PsyCap as a personal resource that mediates the relationship between job crafting and work engagement. By identifying an additional and critical mediating path from job crafting to work engagement, this work contributes to advance our understanding of how job crafters become engaged in their work.

This study also reveals that the extent to which job crafters obtain PsyCap is contingent upon the support that they receive from their coworkers in an organization. In other words, our study demonstrates that coworker support as a job resource plays an essential role in underpinning the relationship between job crafting and PsyCap. In general, it is well known that social support has a great impact on the attitudes and behaviors of organizational members (e.g., [43]). Yet, instead of utilizing a global interpretation of social support, we focused specifically on coworker support for this research on the basis of the differential functions afforded by different sources of social support, and anticipated that not supervisor support but coworker support would serve as a critical boundary condition that links job crafting to PsyCap. This is because a job crafter tends to change his or her task boundaries without the approval of his or her supervisors, and he or she is more frequently exposed to coworkers than to supervisors. In doing so, this study has found that coworker support reinforces the relationship between job crafting and PsyCap, while supervisor support does not. These findings indicate that the important supportive activities for job crafters are not those issuing from their leaders, but rather those that are associated with their colleagues. Further, this work implies that it is desirable to specifically examine the characteristics and recipients of its sources so as to accurately understand the effects of social support.

Lastly, after taking all the factors into consideration, this study proposes a moderated meditation model to fully account for the association between job crafting and work engagement. We have demonstrated that coworker support strengthens the indirect link between job crafting and work engagement through PsyCap. That is, we have found that, when a job crafter receives support from his or her coworkers, he or she is more likely to gain personal resources, which in turn leads to enhanced work engagement. These findings indicate that the impact of job crafting on work engagement might not be as straightforward as assumed to date. Instead, the current study emphasizes the importance of PsyCap between job crafting and work engagement, as well as the meaningfulness of coworker support that fortifies this indirect relationship.

In addition to these theoretical contributions, this study has practical implications for the effective management of business organizations. Given that members’ levels of engagement within an organization determine their health and well-being, as well as individual and organizational performance [2,3,4,6], the findings of this study seem to suggest the value of finding ways to improve the engagement of an organization’s members. First, human resource management (HRM) staff need to inform employees of meanings, contents, and examples of job crafting, so that they alter their tasks on their own. In today’s rapidly changing circumstances, it is not feasible for executives or HRM staff to accurately predict changes and assign tasks to their members [20]. Accordingly, it is now necessary to turn their attention to encouraging voluntary job crafting rather than to executing thorough task allocation.

Meanwhile, it is desirable to formally and informally lead job incumbents to participate in supportive activities amongst themselves. According to the findings of this study, it is not supervisor support but coworker support that plays a decisive role for job crafters in gaining higher levels of PsyCap and engagement at work. In the case of flight attendants, due to the frequent changes of flight schedules [65], immediate supervisors are likely to provide only limited help to their subordinates in the execution of their duties. It is therefore valuable that an organization creates an environment and a culture in which its members are able to help each other and exchange advice. Further, in order to naturally nurture an environment of helping one another, HR staff need to consider adopting an altruistic tendency test during the recruiting process.

### 5.2. Limitations and Future Research Directions

Even if this study provides a research model and the empirical results to comprehensively understand the relationship between job crafting and work engagement, there remain some limitations that should be addressed by future research. Specifically, according to prior studies (e.g., [11]), while job crafting was found to have positive impacts on PsyCap and work engagement, reverse causation effects were not found. We thus believe that job crafting has positive relationships with PsyCap and work engagement although this study was not conducted with longitudinal data. Yet, it was shown that there are reciprocal relationships between personal resources and work engagement [40]. That is, personal resources and work engagement are mutually related, in that personal resources result in enhanced work engagement and then work engagement induces more personal resources. Accordingly, we would expect further research to design a model with a longitudinal perspective.

Additionally, there are limitations regarding the sample included in this study. As described above, we collected our sample from South Korean flight attendants. It has been assumed that the expected relationships between job crafting, PsyCap, coworker support, and work engagement would be generalizable across companies, industries, and nations. It is necessary for future research to replicate and extend this study with more samples incorporating various companies, industries, and nations to secure external validity.

Finally, in this study, we have focused on individual job crafting, namely those employee activities that shape the boundaries of their tasks. Some researchers have argued that job crafting is not only a form of individual job crafting, but also a form of collaborative job crafting [30,66]. Furthermore, it has also been shown that attitudes and performance may vary across different forms of job crafting [64]. Accordingly, in recognition that job crafting might be performed by groups as well as individuals, future research needs to incorporate the two forms of job crafting into the research model to explore the effect of job crafting on work engagement.

## 6. Conclusions

A rapidly changing business environment requires organizations to possess engaged members as an essential condition for their survival and prosperity because employees who are engaged in their work approach their work more energetically, enthusiastically, and engrossedly, and enjoy good mental health. Further, it is recognized that employees’ actions taken to create changes in carrying out tasks in the workplace determine their levels of work engagement. Nonetheless, since little is known about the processes and conditions in which employees who craft their tasks become engaged, we have attempted to identify whether PsyCap mediates and coworker support moderates the relationship between job crafting and work engagement. By revealing how and when job crafters become engaged at work, this study suggests that it is necessary for contemporary organizations to encourage their members to gain more PsyCap that is based on job crafting and to create a mutual cooperative culture in order to enable them to be enthusiastic and healthy.

## Figures and Tables

**Table 1:** Descriptive statistics of variables.

Variables	1	2	3	4	5	6	7	8	9	10	11
1. Gender	-										
2. Age	0.12	-									
3. Education	0.01	0.20 *	-								
4. Job tenure	−0.10	0.74 **	0.15 *	-							
5. Positive affectivity	0.13 ^†^	−0.05	−0.03	−0.03	0.75						
6. Negative affectivity	−0.14 ^†^	−0.10	−0.04	−0.08	−0.36 **	0.59					
7. Job crafting	0.27 **	0.01	−0.08	0.04	0.26 **	−0.11	0.56				
8. PsyCap	0.20 *	0.09	−0.05	0.07	0.40 **	−0.25 **	0.42 **	0.71			
9. Work engagement	0.31 **	0.05	−0.09	0.00	0.57 **	−0.34 **	0.46 **	0.63 **	0.70		
10. Coworker support	0.03	−0.14 ^†^	−0.04	−0.13 ^†^	0.24 **	−0.12	0.07	0.39 **	0.21 **	0.55	
11. Supervisor support	0.07	−0.15 ^†^	−0.10	−0.011	0.36 **	−0.19 *	0.14 ^†^	0.41 **	0.33 **	0.43 **	0.75
Mean	0.11	28.54	15.98	4.90	3.00	2.65	3.11	3.40	3.18	3.68	3.36
SD	0.31	3.87	0.78	4.18	0.80	0.85	0.62	0.43	0.64	0.51	0.63
Cronbach’s α	-	-	-	-	0.90	0.81	0.82	0.85	0.86	0.82	0.89
CR	-	-	-	-	0.90	0.81	0.83	0.91	0.88	0.83	0.90

Note: CR = composite reliability; the number in the diagonal is the AVE. ^†^ *p* < 0.10, * *p* < 0.05, ** *p* < 0.01.

**Table 2:** Psychological capital (PsyCap) and work engagement regressed on job crafting.

Variables	PsyCap			Work Engagement		
	*b*	*se*	*t*	*b*	*se*	*t*
Gender	0.09	0.09	0.99	0.19	0.11	1.76 ^†^
Age	0.01	0.01	1.17	0.01	0.01	0.48
Education	−0.04	0.03	1.17	−0.05	0.04	1.31
Job tenure	0.00	0.01	0.13	−0.00	0.01	0.38
Positive affectivity	0.18	0.04	4.99 **	0.22	0.05	4.64 **
Negative affectivity	−0.06	0.03	1.97 *	−0.09	0.04	2.22 *
Job crafting	0.21	0.05	4.57 **	0.19	0.06	3.28 **
PsyCap				0.57	0.09	6.08 **
*R* ^2^	37.5%			58.9%		

Note: Two-tailed. ^†^ *p* < 0.10, * *p* < 0.05, ** *p* < 0.01.

**Table 3:** Mediating effect of PsyCap.

Effects	*b*	CI_low_	CI_high_
**Total effect**			
Job crafting → Work engagement	0.30	0.16	0.45
**Direct effect**			
Job crafting → Work engagement	0.19	0.06	0.34
**Indirect effect**			
Job crafting → PsyCap → Work engagement	0.12	0.06	0.19

Note: CI = confidence interval (95% level).

**Table 4:** PsyCap regressed on the moderation term of job crafting and coworker support.

Variables	PsyCap		
	*b*	*se*	*t*
Gender	0.08	0.08	0.34
Age	0.02	0.01	1.76 ^†^
Education	−0.04	0.03	1.11
Job tenure	0.00	0.01	0.38
Positive affectivity	0.13	0.04	3.65 **
Negative affectivity	−0.06	0.03	1.79 ^†^
Job crafting	0.18	0.04	4.38 **
Coworker support	0.21	0.05	3.99 **
Supervisor support	0.10	0.04	2.28 *
Job crafting × Coworker support	0.19	0.09	2.11 *
Job crafting × Supervisor support	−0.07	0.05	1.36
*R* ^2^	44.9%		

Note: Two-tailed. ^†^ *p* < 0.10, * *p* < 0.05, ** *p* < 0.01.

**Table 5:** Moderating effect of coworker support.

Levels	*b*	CI_low_	CI_high_
−0.50 (−1 SD)	0.09	−0.06	0.24
0.00 (Mean)	0.18	0.09	0.28
0.50 (+1 SD)	0.28	0.16	0.40

Note: CI = confidence interval (95% level).

**Table 6:** Moderated mediation effect of coworker support.

Levels	*b*	CI_low_	CI_high_
−0.50 (−1 SD)	0.05	−0.03	0.15
0.00 (Mean)	0.10	0.05	0.17
0.50 (+1 SD)	0.16	0.09	0.25

Note: Two-tailed; CI = confidence interval (95% level).
